# Effect of *Agaricus blazei* Murill on the Pulmonary Tissue of Animals with Streptozotocin-Induced Diabetes

**DOI:** 10.1155/2010/543926

**Published:** 2010-05-26

**Authors:** Fábio Cangeri Di Naso, Rodrigo Noronha de Mello, Sílvia Bona, Alexandre Simões Dias, Marilene Porawski, Alexandre de Barros Falcão Ferraz, Marc François Richter, Norma Possa Marroni

**Affiliations:** ^1^Laboratory of Experimental Hepatology and Physiology, Porto Alegre Clinical Hospital, Federal University of Rio Grande do Sul, 90035-903 Porto Alegre, RS, Brazil; ^2^Universidade Luterana do Brasil, 92425-900 Canoas, RS, Brazil; ^3^Centro Universitário Metodista IPA, 90240-111 Porto Alegre, RS, Brazil; ^4^Universidade Federal de Ciências da Saúde de Porto Alegre, 90050-170 Porto Alegre, RS, Brazil; ^5^Universidade Estadual do Rio Grande do Sul, 90010-191 Porto Alegre, RS, Brazil

## Abstract

The present study was designed to evaluate the oxidative stress as well as the therapeutic effect of *Agaricus blazei* Muril (*A. Blazei*) in rats with streptozotocin-induced diabetes. We used 25 Wistar rats, and DM was induced by injecting streptozotocin (70 mg/Kg i.p.). *Agaricus blazei* Muril was administered daily starting 40 days after disease onset. *A. Blazei* was tested as an aqueous extract for its phytochemical composition, and its antioxidant activity in vitro was also evaluated. Lipoperoxidation (LPO), and superoxide dismutase (SOD), catalase, and glutathione peroxidase activities were measured in the pulmonary tissue, as well as the presence of inducible nitric oxide synthase (iNOS), through immunohistochemistry. An anatomopathologic study was also performed. Phytochemical screening of *A. Blazei* detected the presence of alkaloids and saponins. The extract exhibited a significant antioxidant activity in the DPPH-scavenging and the hipoxanthine/xanthine oxidase assays. Pulmonary LPO increased in diabetic animals (0.43 ± 0.09; *P* < .001) as compared to the control group (0.18 ± 0.02), followed by a reduction in the *A. Blazei*-treated group (0.33 ± 0.04; *P* < .05). iNOS was found increased in the lung in diabetic rats and reduced in the *A. Blazei*-treated group. The pulmonary tissue in diabetic rats showed oxidative alterations related to the streptozotocin treatment. The *A. Blazei* treatment effectively reduced the oxidative stress and contributed to tissue recovery.

## 1. Introduction

*Diabetes mellitus* (DM) is an endocrine metabolic disease of growing incidence and clinical relevance with high morbidity and mortality rates [1]. Among its chronic complications are the micro- and macro vascular disorders related to the renal, cardiovascular, and nervous systems [2]. However, in the last two decades, changes in the respiratory function have also been reported in clinical and experimental studies. Decreases in the pulmonary function over the years, related to the decreased measures of pulmonary volumes and capacity, were evidenced in diabetic patients with impaired metabolic control [3, 4]. Structural alterations to the basal membrane of the pulmonary capillary endothelium are also present in DM, with a thickening of the alveolus-capillary membrane and reduction in the diffusional capability [5, 6]. Moreover, diabetic patients are more susceptible to lung infections, in particular tuberculosis, which has a four-times greater incidence in this particular population [7, 8]. Although all these alterations were evidenced in clinical and experimental studies, few studies investigated the main physiopathologic mechanisms involving pulmonary complications related to DM. 

There are 4 pathways associated with chronic complications of DM, namely, the polyol pathway, protein kinase C (PKC) activation, increased flow in the hexosamine pathway, and the pathway of advanced glycosilation end-products (AGE). Although presenting differently in each case, oxidative stress (OS) is implicated in the four pathways cited above [9]. 

There is plenty of evidence showing that the increase of nitric oxide (NO), formed by the action of inducible nitric oxide synthase (iNOS) is one of the factors responsible for both the pathogenesis and the complications resulting from DM [10, 11]. The use of exogenous antioxidants may represent a great therapeutic potential for treatment of DM [12–14]

The basidiomycete *Agaricus blazei* Murill (*A. Blazei*), popularly known as “sun mushroom”, is native to Brazil and widely grown in Japan because of its medicinal properties. This mushroom is traditionally used in the treatment of atherosclerosis, hepatitis, hyperlipidemia, dermatitis, and cancer, and it has been shown to have immunomodulating and antimutagenic effects both in vivo and in vitro. Polysaccharides *α*-glycan and *β*-glycan are responsible for the function of immunological and antitumoral stimulation [15–17].

*A. Blazei* has already been shown to be beneficial in insulin resistance related to type 2 diabetes, but no study has shown the antioxidant potential of *A. Blazei* in vivo in DM [17]. Thus, this study was designed to evaluate the oxidative stress as well as the therapeutic effect of *A. Blazei* in the pulmonary tissue of animals with streptozotocin-induced DM. 

## 2. Methods

### 2.1. Mushrooms

Air-dried mushrooms of the species *Agaricus blazei* Murill (C type) were a gift from Dr. Luiz Antônio Graciollo, Department of Engeneering at the State University of São Paulo (UNESP), Brazil. 

### 2.2. Preparation of *A. blazei* Aqueous Extract

Air-dried parts (100 g) were milled and the aqueous extracted was prepared by infusion (1/10 mushroom/solvent). The infusion stood at room temperature for 30 minutes. After cooling and filtration, the extract was frozen and concentrated by lyophilization for five days overnight, in order to obtain the *A. blazei *aqueous extract. 

### 2.3. Chemicals and Reagents

2,2-Diphenil-1-picrylhidrazyl (DPPH), hypoxanthine, xanthine oxidase, trolox, and salicylic acid were purchased from Sigma (St. Louis, USA).

### 2.4. Phytochemical Screening

The phytochemical analysis (flavonoids, tannins, anthraquinones, alkaloids, saponins, coumarins and cardiac glycosides) of *A. blazei* was carried out according to the methods described by Harborne [18]. The thin layer chromatography analyses were performed following systems and developers indicated by Wagner and Bladt [19].

### 2.5. Hypoxanthine/Xanthine Oxidase Assay

The method employed to assay the hydroxyl radical scavenging ability of the extracts was based on the method of Owen et al. [20]. Briefly, the extract was dissolved in the assay buffer (hypoxanthine, Fe(III), EDTA and salicylic acid) at a concentration of 2.0 mg/mL and diluted appropriately (in triplicate) in assay buffer to a final volume of 1.0 mL giving a range of 0.1–2.0 mg/mL. A 5 *μ*L aliquot of xanthine oxidase dissolved in 3.2 M (NH_4_)_2_SO_4_ was added to initiate the reaction. The sample tubes were incubated for 3 hours at 37°C, at which time the reaction was complete. A 30 *μ*L aliquot of the reaction mixture was analyzed by HPLC using chromatographic conditions as described by Owen et al. [21, 22]. Chromatographic analysis was done using a gradient based on methanol/water/acetic acid with a *μ*BondaPak C18 reverse phase column and detection at 325 nm. The HPLC equipment had a 2695 separation module and UV detector 2487. The hydroxylation of salicylic acid and hypoxanthine were monitored at A = 325 and A = 278 nm, respectively. The amount of dihydroxyphenols (2,5-dihydroxibenzoic acid and 2,3-dihydroxibenzoic acid) (2,5-DHBA and 2,3-DHBA) produced by hydroxyl radical (OH•) attack on salicylic acid was determined from standard curves prepared with the respective pure dihydroxyphenols.

### 2.6. DPPH-Scavenging Assay

Scavenging of DPPH free radical was measured using a modified method described by Yamaguchi et al. [23] in which the different methanolic plant extracts were added to Tris–HCl (100 mM) buffer, pH 7.0, containing 250 mM DPPH dissolved in methanol. At least six different dilutions of each extract were tested, and allowed to stand 20 minutes in the dark, before absorbance was measured at 517 nm using a Shimadzu spectrophotometer model UV-1602PC (Kyoto, Japan). The experiment was conducted in triplicate. Antioxidant activity (AOA) was expressed as IC_50_ (inhibitory concentration in *μ*g/mL of samples or positive controls necessary to reduce the absorbance of DPPH by 50% compared to the negative control). The lower the IC_50_, the higher is the AOA [23]. 

### 2.7. Animals and Experimental Protocol

The experimental protocol used complied with the norms established by the Ethical and Health Research Committee of the Group of Research and Postgraduate Studies of the Hospital de Clínicas of Porto Alegre as well as with the *Principles for Research Involving Animals *(NAS). Only male Wistar rats were used, obtained from the breeding colony of the Instituto de Ciências Básicas da Saúde da Universidade Federal do Rio Grande do Sul (UFRGS). The mean weight of animals at the start of the study was 200–300 grams. They were kept under a 12 : 12 hours light/dark cycle (light from 7 a.m. to 7 p.m.) in a temperature-controlled environment (22 ± 4°C).

DM was induced by a single injection of streptozotocin i.p. (STZ, Sigma *Chemical Company*, *St. Louis*, MO, EUA) at a dose of 70 mg/Kg of body weight [24]. STZ was dissolved in sodium citrate buffer (0.1 M, pH 4.5) and administered in the left abdominal region of the animal about 10 minutes after dissolution in the buffer solution. The animals in the control group received only NaCl 0.9% i.p. at the same volume of the buffer used to dissolve STZ. The *A. blazei* extract was diluted to the concentration of 0.1 g/mL (10%) in a solution of distilled water and left for 2 hours at room temperature [25]. The administration route was gastric gavage with a final solution of 2 mL and treatment was initiated from the 40th day of diabetes induction. The animals were randomized in the different groups: control (CO), diabetic treated with NaCl (DM), and diabetic treated with *A. blazei* (DM + *A. blazei*). Blood samples were collected from the retro-orbital plexus one day before induction, and 2 and 30 days after the beginning of the experiment. At the end of the 60 days of trial the animals was induce to euthanasia by exsanguination, after anesthetized with xilasine and ketamine. Blood from the retro-orbital plexus was sampled and the right lung was dissected out and kept in 4% formaldehyde for histological analysis. The left lung was removed and frozen at −80°C for additional analyses. 

### 2.8. Serum Analyses

The blood samples were placed into a testing tube with heparin (Liquemine) to avoid coagulation. The material was then centrifuged at 1.800 × g for 15 minutes. The precipitate was discarded and the plasma removed. 

To determine glucose, cholesterol and triglycerides levels we used the colorimetric enzymatic test (Kit Labtest, Bio Diagnóstica) and absorbance was measured in spectrophotometer (CARY 3E-UV-*Visible Spectrophotometer Varian*). Animals with a glucose concentration above 250 mg/dL [26] were considered as diabetic. 

### 2.9. Biochemical Analyses of Oxidative Stress and Antioxidant Assay

The lungs were homogenized with 9 mL of phosphate buffer (KCL 140 mM, phosphate 20 mM, pH 7.4) per gram of tissue. The protein concentration in these lung homogenates was determined using a standard solution of bovine albumin according to Lowry et al. [27].

Pulmonary lipoperoxidation was determined by the method of thiobarbituric acid reactive substances (TBA-RS) [28].

Superoxide dismutase (SOD) activity in the lung tissue was determined using a technique based on the inhibition of adrenochrome formation in epinephrine autoxidation [29]. Catalase (CAT) activity in the lung tissue was determined as described elsewhere [30] and the determination of selenium-dependent glutathione peroxidase in the lung tissue was obtained through a technique consisting in the measure of NADPH oxidation by glutathione reductase [31].

### 2.10. Histological Study

For the histological analysis the samples were embedded in paraffin twice. Using a microtome, the paraffin blocks were cut into 3-*μ*m seriate sections. In the staining phase, the slides were immersed in hematoxylin-eosin and picrosirius. In the dehydration phase, the structures went through three containers with absolute alcohol and two containers with xylol. Reading was performed with light microscopy (*Nikon Labophot*) at 100×. The analysis was performed by 2 pathologists who did not know the study details. 

### 2.11. Immunohistochemical Detection of iNOS

Immunohistochemical reactions were performed in the lung tissue sections through the technique of streptavidin-biotin peroxidase complex (StreptABC, DAKO). The slides were previously coated by a silane solution (APTS, Sigma) diluted in 4% acetone. 3-*μ*m thick sections were obtained using a mechanical microtome. The sections were then deparaffinized and successively immersed in xylol and ethanol and submitted to antigenic recovery by irradiation heat in pressure cooker (Eterna, Nigro) using citrate buffer (10 mM, pH 6.0) for 15 minutes. Peroxidase blocking was performed using a hydrogen peroxide solution at 3%, followed by incubation with primary antibody against NOS-2 (iNOS, 1 : 40, Santa Cruz). The reactions were marked with diaminobenzidine (DAB, Sigma) solution at 60 mg% and counterstained with Harris's hematoxylin (Merck). For each reaction a positive control was used to tissue that was known to be positive for the tested antibody. Two negative controls were also used, the first one by absence of the primary antibody and the second by removing the secondary antibody during the reaction steps. The cases were considered as iNOS-positive when the brown coloration of at least moderate intensity was visible in the cell cytoplasm and in more than 10% of the cells. 

### 2.12. Statistical Analysis

The data are presented as mean ± standard deviation (SD) and were analyzed through statistical software SPSS 15.0. The variables were tested for normality through the Kolmogorov-Smirnov test. One-way analysis of variance (ANOVA) was used for intergroup differences. Student Newman-Keuls post hoc test was used for parametric variables and Kruskal-Wallis for the nonparametric ones. The level of significance used was *P* < .05. 

## 3. Results

### 3.1. Phytochemical Analyses

Phytochemical analyses of *A. blazei* indicated the presence of saponins and alkaloids. Other secondary metabolites such as anthraquinones, cardiac glycosides, cumarins, flavonoids, fenolic acids and tannins were not detected. 

### 3.2. Hypoxanthine/Xanthine Oxidase In Vitro Assay

**Figure 1:**
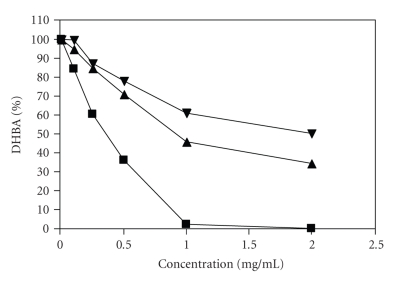
Inhibition of the generation of reactive oxygen species by aqueous extracts of aerial parts of *Agaricus blazei* (▴), *Lentinula edodes* (▾) and Trolox, used as positive control (*▪*) using the hypoxanthine/xanthine oxidase system. Data points are presented as mean of ± SD, *n* = 3.

The in vitro antioxidant activity of the extract was determined by monitoring the production of hydroxyl benzoic acids (DHBA) as a product of the hydroxyl radical attack on salicylic acid in the hipoxanthine-xanthine oxidase assay. The reduction of total oxidation products as a function of the concentration of *A. blazei* aqueous extract added to the assay resulted in an in vitro antioxidant capacity in a dose-dependent manner. The aqueous extract of *A. blazei* reduced the formation of both DHBA species to 45.2 % in the highest concentration used (2 mg/mL). The IC_50_ value was calculated and found to be 0.99 mg/mL. A second type of mushroom (*Lentinula edodes*) for which the authors did not find the presence of alkaloids was used as control sample (IC_50_ of 1.95 mg/mL). Trolox (vitamin E) was used as positive control and displayed an IC_50_ of 0.34 mg/mL (Figure 1).

### 3.3. DPPH-Scavenging Assay

The free radical scavenging effect of the both of *A. blazei *aqueous extract, *L. edodoes* aqueous extract, as well as trolox, as positive control, was tested, using the DPPH free radical scavenging assay [23]. The IC_50_ values for *A. blazei *aqueous extract and for *L. edodes* extract are shown in Table 1. The results of the free radical scavenging effect of trolox (IC_50_ = 0.02 mg/mL), used as positive control, was used to validate the assay. Although the free radical scavenging capacities of the extracts was lower (higher concentration are necessary to reduce the absorbance of DPPH by 50%) than the effect of trolox, the *A. blazei *aqueous extract (with highest flavanone content) presented promising antioxidant activity with an IC_50_ of 1.77 mg/mL. *L. edodes*, on the other hand, had the lowest scavenging activity (IC_50_ = 3.22 mg/mL) which is in agreement with the absence of alckloids in this species of mushroom.

### 3.4. Body Weight and Serum Analyses

The body weight of diabetic animals was significantly reduced, and *A. Blazei*-treated animals lost weight still more (Table 2). The *A. Blazei* extract apparently reduced glycemia in the diabetic rats (*P* < .01) but the glycemic curve was similar across diabetic and treated animals. However, *A. Blazei* significantly reduced the total cholesterol and triglycerides levels (*P* < .01). The DM and DM + *A. Blazei* groups had different sample sizes due the higher mortality of the animals of DM group during the experiment.

### 3.5. Biochemical Analysis and Oxidative Stress

*A. Blazei* significantly reduced (*P* < .05) the lipoperoxidation levels as determined by TBA-RS (Table 3). However, activity of antioxidant enzymes SOD and CAT did not show any differences between the groups. Enzyme GPx activity was significantly increased in the diabetic group and reduced in the *A. Blazei*-treated group (*P* < .05).

### 3.6. Histological Analysis

**Figure 2:**
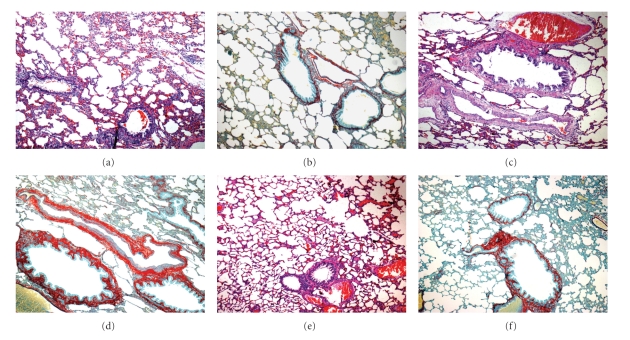
Histology of lung tissue stained by HE (a, c and e) and picrosirius (b, d and f). Magnification 100×: (a) and (b): Control, (c) and (d): Diabetes Mellitus, (e) and (f): Diabetes Mellitus treated with *Agaricus blazei*.

STZ-induced Diabetes Mellitus caused serious vascular injury to the pulmonary tissue (Figure 2(c)), where alveolar changes such as septa rupture were also evidenced. Picrosirius staining revealed an expansion of the conjunctive tissue in the alveolocapillary space of the diabetic group (Figure 2(d)) and an apparent reversion of this pattern in the Ab-treated group (Figure 2(f)).

### 3.7. Immunohistochemical Analysis of iNOS

**Figure 3:**
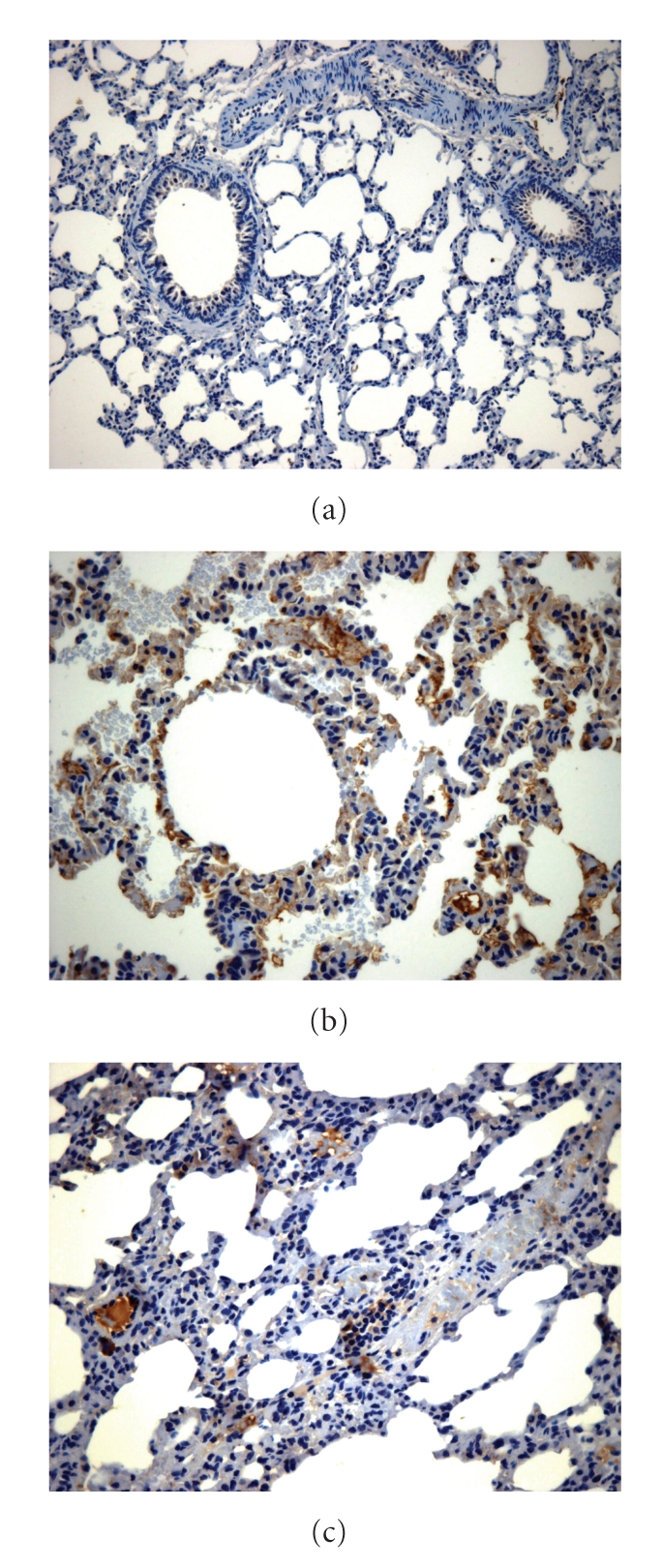
iNOS immunohistochemistry in lung tissue. Magnification 400×: There was no staining in the control group (a); reduction in the treated group (b) versus DM (c).

Figure 3 shows iNOS distribution in the lung tissue as detected through immunohistochemistry. The positive stain in brown seen in the pulmonary bronchial epithelium and capillary endothelium in the DM group indicated iNOS positivity. iNOS staining was less apparent in the *A. Blazei* group and absent in the CO group. 

## 4. Discussion

Results of the free radical scavenging effect of the *A. blazei* aqueous extract in the hipoxantine/xanthine oxidase in vitro assay, and in the DPPH free radical scavenging assay, showed a significant in vitro antioxidant activity. In both assays the extract expressed a higher antioxidant activity, in comparison with the aqueous extract of *L. edodes*, which is another species of mushroom and which presents only saponins, but not alkaloids, or flavonoids or tannins. It has been suggested by Ribeiro et al. that the antioxidant activity might be related to the presence of alkaloids in the mushroom. In other words, higher alkaloid concentrations generate better antioxidant activity [32]. 

The main finding of this study was the reduction of pulmonary lipoperoxidation in rats with streptozotocin-induced diabetes after treatment with *Agaricus blazei*. Previous studies have shown a glycemia-reducing effect, which decreases insulin resistance and enhances the release of it by *β*-pancreatic cells [17, 33]. However, *A. Blazei* treatment here showed a beneficial effect regarding the variables related to the oxidative stress, despite not reducing hyperglycemia. 

Kim et al. described antidiabetogenic effects of *β*-glucanes extracted from *A. Blazei* and its enzymatically hydrolyzed oligosacharides, evaluating the in vitro and in vivo effects in culture of *β*-pancreatic cells and in animals with streptozotocin-induced diabetes. After treatment with *β*-glucanes and oligosacharide the animals presented reductions in glycemia, triglycerides, and cholesterol levels and in atherosclerotic activity [34]. In our study, the treatment was performed using a gross extract of *A. Blazei*, without isolating any of its compounds, and this is probably the explanation for the lack of antiglycemic action.

The extract of *A. Blazei* demonstrated in vitro and in vivo antioxidant activity, however the treatment significantly reduced the weight of the animals. This fact could be explained due to high doses of *A. blazei *extract used in this experiment, differently of doses used in others studies that do not demonstrate weight reduction [34]. However, in a study to evaluate 90-day subchronic toxicity of an aqueous extract in rats, there were no consistent treatment-related changes in clinical signs, body weight and food consumption at the dose of 2654 mg kg^−1^ for male rat, a higher dose than used in our study [15]. More studies were necessary to evaluate the toxic effect of A. *Blazei *in these doses, with the analysis of specific variables. 

GPx was significantly increased in the diabetic group and was significantly reduced after *A. Blazei* treatment. This increase in GPx can account for the diminished levels of reduced glutathione, as it is the main substrate regulating its activity. Gumieniczek et al. demonstrated that in experimental DM the pulmonary oxidative stress is present because of the reduction of antioxidant enzyme activity and increased lipoperoxidation. Such changes are more significant after weeks following induction. During DM there is a decrease of Cu,Zn-SOD activity and an increase of catalase activity [35]. In our study, neither SOD nor catalase activity were changed in none of the different groups. A possible explanation for our findings differing from those of Gumieniczek is that in our study the analysis of antioxidant enzymes was performed earlier. 

In our experimental model numerous histological alterations were observed in the pulmonary system. These alterations are in agreement with those reported in the literature [7, 36, 37], especially as regards the increase in the conjunctive tissue and thickening of the basal lamina observed through the technique of picrosirius staining. After treatment with *A. Blazei* such alterations became less evident. The formation of intra-and intermolecular binding with collagen, resulting from the process of glycosilation, leads to structural alterations in tissue proteins such as increase in rigidity, resistance to proteolytic digestion and the extracellular matrix (including fibronectin, procollagen **α**2, type III, IV and VI collagen, and laminina) [38, 39]. In our study, the main factor for the reversion of this process after treatment with *A. Blazei* can be explained by the reduction of damage resulting from the oxidative stress demonstrated by the reduction of pulmonary lipoperoxidation. 

A long term hyperglycemic state is related to alterations in iNOS expression in several tissues [10]. In the immunohistochemical analysis of our study iNOS was significantly increased in the pulmonary tissue of the diabetic rats and significantly decreased when the animals were treated with *A. Blazei*. Studies have demonstrated that mRNA expression of endothelial nitric oxide synthase (eNOS) is reduced, whereas iNOS may be increased together with the generation of cyclic guanosine monophosphate (c-GMP) [14].

In a recently published study, lipoperoxidation, superoxide dismutase activity, and the distribution of iNOS and eNOS isoforms were evaluated in the lung tissue of diabetic rats. An increase in the oxidative stress concomitant with the increased iNOS in the lung tissue of diabetic rats was observed, which was reversed in the group treated with antioxidant *α*-lipoic acid [40]. These findings are in agreement with those reported in the present work, such as the observed increased oxidative stress, the histological pulmonary alterations, and the effect of an antioxidant therapy in this DM model. 

The present study demonstrates the beneficial effect of *A. Blazei* aqueous extract regarding oxidative stress variables and pulmonary morphopathology in streptozotocin-induced diabetes. These findings may contribute significantly to a better understanding of the pulmonary physiopathology in DM. Our study is also relevant and with regard to the therapeutic potential of *A. Blazei*. 

## Figures and Tables

**Table 1:** Inhibition of DPPH*, IC_50_ values for the DPPH assay of aqueous extracts of *Agaricus blazei* and *Lentinula edodes* mushrooms, and trolox.

Sample	Inhibition of DPPH (%)					
Concentration	0.1 *μ*g/mL	0.25 mg/mL	0.5 mg/mL	1 mg/mL	2 mg/mL	IC_50_ (mg/mL)
Trolox	91.27	93.53	96.71	99.03	99.89	0.02 ± 0.00
*Agaricus blazei*	7.28	10.77	17.09	46.32	48.81	1.77 ± 0.08
*Lentinula edodes*	2.19	4.68	8.33	18.56	30.63	3.22 ± 0.12

Mean ± standard deviation of three individual determinations. Results were based on the values measured at 20 minutes. Trolox was used as positive control. *DPPH: 2,2-diphenyl-1-picrylhydrazyl.

**Table 2:** Changes in body weight and glucose, cholesterol and triglycerides plasma levels.

	*N*	Weight (g)	Glucose (mg/dL)	Total Cholesterol (mg/dL)	Triglycerides (mg/dL)
CO	5	442.00 ± 10.95	244.17 ± 68.01	28.35 ± 4.62	61.33 ± 33.43
DM	8	306.22 ± 32.11^†^	482.37 ± 36.81*	42.88 ± 6.44*	161.00 ± 76.80^##^
DM + *A. Blazei *	12	282.00 ± 44.11^#^	468.19 ± 62.46^#^	33.99 ± 5.23**	45.87 ± 10.61**

Data appear as mean ± SD. CO: Control, DM: Diabetes Mellitus and DM + *A. Blazei*: Diabetes Mellitus+ *Agaricus blazei.*

^†^ *P* < .001 CO versus DM.

^#^ *P* < .05 DM versus DM + *A. Blazei.*

*P < .01 CO versus DM.

**P < .01, DM versus DM + *A. Blazei.*

^##^ *P* < .05 CO versus DM.

**Table 3:** Biochemical analyses of oxidative stress in lung tissue.

	TBARS (nmoles/mg of protein)	SOD (U/mg de proteín)	CAT (pmoles/mg de protein)	GPx (nmoles/mg de protein)
CO	0.18 ± 0.02	76.33 ± 3.39	0.10 ± 0.04	0.41 ± 0.07
DM	0.43 ± 0.09*	69.32 ± 11.73	0.18 ± 0.07	1.10 ± 0.53*
DM + *A. Blazei *	0.33 ± 0.04**	74.84 ± 8.75	0.15 ± 0.03	0.45 ± 0.09**

Data appear as mean ± SD. CO: Control, DM: Diabetes Mellitus and DM + *A. Blazei*: Diabetes Mellitus+ *Agaricus blazei.*

*P < .01 CO versus DM.

**P < .05 DM versus DM + *A. Blazei.*
